# Pharmacological Regulation of Oxidative Stress in Stem Cells

**DOI:** 10.1155/2018/4081890

**Published:** 2018-09-30

**Authors:** Jungwoon Lee, Yee Sook Cho, Haiyoung Jung, Inpyo Choi

**Affiliations:** ^1^Immunotherapy Convergence Research Center, Korea Research Institute of Bioscience and Biotechnology (KRIBB), 125 Gwahak-ro, Yuseong-gu, Daejeon 34141, Republic of Korea; ^2^Department of Bioscience, University of Science and Technology (UST), 113 Gwahak-ro, Yuseong-gu, Daejeon 34113, Republic of Korea; ^3^Department of Functional Genomics, University of Science and Technology (UST), 113 Gwahak-ro, Yuseong-gu, Daejeon 34113, Republic of Korea

## Abstract

Oxidative stress results from an imbalance between reactive oxygen species (ROS) production and antioxidant defense mechanisms. The regulation of stem cell self-renewal and differentiation is crucial for early development and tissue homeostasis. Recent reports have suggested that the balance between self-renewal and differentiation is regulated by the cellular oxidation-reduction (redox) state; therefore, the study of ROS regulation in regenerative medicine has emerged to develop protocols for regulating appropriate stem cell differentiation and maintenance for clinical applications. In this review, we introduce the defined roles of oxidative stress in pluripotent stem cells (PSCs) and hematopoietic stem cells (HSCs) and discuss the potential applications of pharmacological approaches for regulating oxidative stress in regenerative medicine.

## 1. Introduction

**Figure 1:**
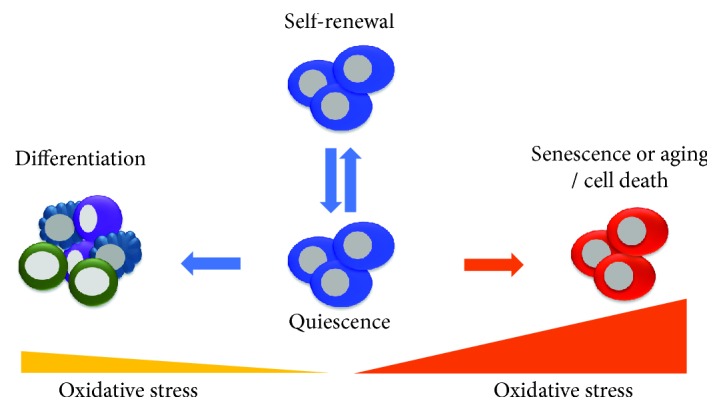
The impact of oxidative stress on stem cells. Quiescent and self-renewing stem cells maintain low ROS level and reside in hypoxic environment. Mild increase of ROS in stem cells causes lineage differentiation; however, acute or excessive ROS cause stem cell senescence or aging and cell death.

Reactive oxygen species (ROS) are originally thought to be a harmful byproduct that is produced intracellularly through aerobic metabolism in the mitochondria [1, 2]. However, recent studies have suggested that ROS regulate physiological and biological functions in cellular processes [3]. ROS are tightly regulated by antioxidant enzymes and modulators under normal physiological conditions. Excessive ROS accumulation occurs in certain conditions and thus makes detoxification beyond the capacity of the antioxidant cellular defense system difficult [4, 5]. Oxidative stress resulting from excessive ROS production and impaired antioxidant systems can affect proliferation, differentiation, genomic mutations, aging, and stem cell death [3, 6–8]. The balance between stem cell self-renewal and differentiation is critical for tissue homeostasis throughout an organism's lifespan, and recent embryonic and adult stem cell reports have shown that this balance is regulated by ROS [2]. Thus, the regulation of the redox state is important for maintaining the function of stem cells and is critical for the fate decision of stem cells (Figure 1).

In regenerative medicine, stem cells are developed to replace damaged tissues; therefore, the appropriate differentiation and maintenance of stem cells are crucial processes for clinical applications. The regulatory mechanisms of oxidative stress and the redox state should be fully defined before stem cells are used in clinical trials. To regulate oxidative stress in stem cells, many research groups have found critical signaling pathways and have suggested their own pharmacologic approaches for mediating them. Therefore, we will review the function, critical signaling pathways, and pharmacological regulation of oxidative stress in pluripotent stem cells (PSCs) and hematopoietic stem cells (HSCs).

## 2. Oxidative Stress in Pluripotent Stem Cells

**Figure 2:**
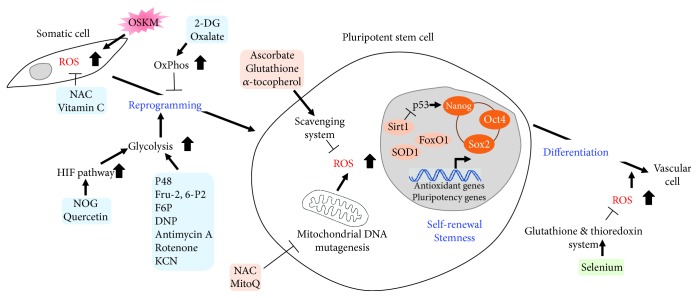
Pharmacological regulation of oxidative stress in PSCs. Forced transduction of OSKM reprogramming factors increases ROS levels which causes DNA damage and inhibits somatic cellular reprogramming into iPSCs. Antioxidants are able to improve reprogramming efficiency and genome stability by quenching ROS levels. During somatic cellular reprogramming, metabolic shift from OxPhos to glycolysis can be modified by different antioxidants, thereby affects the efficient iPSC generation. PSCs are highly sensitive to oxidative stress and affected by the fine control of antioxidants for the maintenance and enhancement of PSC functions as well as the differentiation toward vascular lineage. Oct4, Sox2, Klf4, and c-Myc (OSKM); N-acetyl-L-cysteine (NAC); 2-deoxyglucose (2-DG); fructose 2,6-bisphosphate (Fru-2,6-P_2_); fructose 6-phosphate (F6P); 2,4-dinitrophenol (DNP); N-oxaloylglycine (NOG); mitochondria-targeted ubiquinone (MitoQ).

PSCs, including embryonic stem cells (ESCs) and induced pluripotent stem cells (iPSCs), have the unique properties of undergoing infinite self-renewal and retaining pluripotency to differentiate into every cell type in the body; thus, PSCs represent a valuable source of cells for applications in regenerative medicine [9]. The balance between stem cell self-renewal and differentiation is critical for the developmental process and tissue homeostasis [4]. Recent studies have shown that this manipulation of stem cell fate is partially regulated by ROS, which mediate the oxidation-reduction (redox) state of cells as a secondary messenger [2, 4]. Low ROS levels are necessary for the maintenance of PSCs, whereas oxidative stress due to increased ROS production and damaged ROS scavenging systems can lead to genomic instability, differentiation, death, and/or PSC aging [2]. Here, we introduce the signaling pathways, significant roles and functions of ROS, and the pharmacological regulation of oxidative stress in PSC stemness, pluripotency, and reprogramming (Figure 2).

### 2.1. Oxidative Stress in Stemness

At the early embryo developmental stages, ESCs reside in a hypoxic microenvironment, where the cells use glycolysis to quickly produce very low levels of ATP; however, during the differentiation process, ATP production increases via oxidative phosphorylation (OxPhos), which in turn generates ROS [10]. Thus, it is not surprising that PSCs have the unique features of only a few mitochondria with immature morphology, low oxygen consumption, upregulated glycolytic or antioxidant enzymes, and a shortened G1 cell cycle phase [2, 5], which allow for rapid proliferation, DNA replication, and biomass reproduction compared with typically quiescent differentiated cells [11].

PSCs are sensitive to H_2_O_2_-induced senescence, and they enter a transient G2/M cell cycle arrest state with self-renewal capacity [12]. In addition, PSCs sustain clonal recovery, genomic integrity [13], and pluripotency [14] when cultured in hypoxic conditions. Stemness feature of PSCs is especially sensitive to subtle changes in ROS signaling, originating from mitochondrial DNA (mtDNA) mutagenesis which is associated with an increase in mitochondrial H_2_O_2_. Two different antioxidants, N-acetyl-L-cysteine (NAC) and mitochondria-targeted ubiquinone (MitoQ), efficiently rescue and improve PSC stemness, indicating that PSC functions are modulated by mitochondrial ROS levels [15, 16]. Interestingly, the low-dose components of an antioxidant cocktail (ascorbate, glutathione, and *α*-tocopherol) also affect the free-radical scavenging activity and in turn improve the quality and stability of PSCs; however, high-dose antioxidants which result in an extreme suppression of ROS level downregulate the DNA repair-related kinases and conversely cause the genomic instability of PSCs [17] (Figure 2). Therefore, PSCs are highly sensitive to oxidative stress and affected by the fine control of antioxidants.

### 2.2. Oxidative Stress in Pluripotency

The metabolic shifts between glycolysis and OxPhos are accompanied by the differentiation of PSCs [4]. The enhancement of glycolysis via hypoxia and the suppression of OxPhos, which lead to concomitantly decreased ROS levels, promote the maintenance and proliferation of PSCs, thereby repressing differentiation [14, 18]. Endogenous ROS levels are increased by the sirtuin 1- (SIRT1-) mediated inhibition of p53's antioxidant function. SIRT1, a longevity-promoting NAD^+^-dependent class III histone deacetylase, is also involved in PSC functions by regulating the p53-dependent expression of the pluripotency marker Nanog [19]. SIRT1 is suppressed precisely during human PSC differentiation, resulting in the reactivation of developmental genes, such as the neuroretinal morphogenesis regulators DLL4, TBX3, and PAX6 [20]. Another cellular antioxidant regulator, forkhead box O 1 (FoxO1), is essential for maintaining human ESC pluripotency mediated by the direct activation of octamer-binding transcription factor 4 (Oct4) and sex-determining region Y-box 2 (Sox2), which regulate the circuit of pluripotency [21]. Similarly, superoxide dismutase 1 (Sod1) is also modulated by Oct4, Sox2, and Nanog, suggesting a core relationship between redox homeostasis and pluripotency in PSCs [22].

Conversely, the forced activation of OxPhos led to the loss of stem cell properties and increased differentiation changes. For example, uncoupling protein 2 (UCP2), which is a gatekeeper for the oxidation of carbon substrates, plays an important role in regulating PSC metabolism and differentiation [23]. To achieve differentiation into functional cardiomyocytes, PSCs must be converted to preferentially use the more efficient mitochondrial-mediated oxidative metabolism. In particular, mitochondrial-dependent energetic circuits are key regulators of cardiogenesis and heart regeneration [4, 24]. These marked metabolic differences between PSCs and cardiomyocytes facilitate the large-scale purification of cardiomyocytes from PSCs because culture with glucose-depleted medium containing abundant lactate results in only cardiomyocyte survival [25]. In addition, PSC differentiation toward vascular smooth muscle cells (VSMCs) has been shown to be dependent on the H_2_O_2_ signaling induced by the upregulation of NADPH oxidase 4 (Nox4), which contributes to the production of ROS [26]. The redox function of apurinic/apyrimidinic (AP) endonuclease 1/redox factor 1 (Ape/Ref1) is also critical for mouse ESC differentiation towards the hematopoietic lineage [27], and thioredoxin (Trx) is involved in the regulation of Oct4 activity [28]. Selenium, which enhances antioxidant activities of the glutathione and Trx systems, is able to reduce increased ROS production by Nox4 moderately, thereby promoting the vascular differentiation of human ESCs [29] (Figure 2). Taken together, the decision of PSC fate may be regulated directly by the cellular redox state, which is influenced by PSC metabolic shifts.

### 2.3. Oxidative Stress in Somatic Reprogramming

Somatic cellular reprogramming into iPSCs by the forced transduction of a combination of defined reprogramming factors, namely, Oct4, Sox2, kruppel-like factor 4 (Klf4), and c-Myc (OSKM, named as the “Yamanaka factors”), is a major technological breakthrough in stem cell biology and regenerative medicine; this breakthrough provides a way to produce patient-specific personalized PSCs [30, 31]. However, concerns remain regarding technical issues, including the low efficiency and safety of iPSC generation for their application for therapeutic use.

Similar to the early embryogenesis that occurs in hypoxic niches, hypoxic conditions, which increase glycolysis, play an important role in somatic cellular reprogramming. In this way, the efficiency of mouse and human iPSC generation is higher in hypoxic conditions (1% and 5% O_2_) than in normoxic (21% O_2_) conditions. Moreover, iPSC generation is achieved with only two of the four factors (Oct4 and Klf4) when cultured in hypoxic conditions [5, 32]. Hypoxia-inducible factors (HIFs) regulate not only glycolysis-related genes, such as pyruvate dehydrogenase kinase-1 (PDK1), lactate dehydrogenase (LDH), and glycogen phosphorylase liver (PYGL) [33], but also transcriptional networks that control stemness, such as Oct4, Sox2, and Nanog, which all are associated with somatic cellular reprogramming [5]. In particular, HIF-2*α*, but not HIF-1*α*, binds directly to predicted hypoxic response elements (HREs) in the proximal promoters of Oct4, Sox2, and Nanog in human PSCs under only hypoxia (5% O_2_) conditions; in this way, HIF-2*α* helps regulate the function of PSCs [34, 35]. These findings suggest that hypoxic conditions enhance induced pluripotency, consistent with the responses observed for PSC phenotypes.

During somatic cellular reprogramming, OSKM reprogramming factor-transduced cells have substantially elevated ROS and oxidative stress levels both *in vitro* [36, 37] and *in vivo* [38, 39]. ROS are also produced by metabolic stress and increased ROS levels then lead to cell damage, senescence, and apoptosis. The survival rate of reprogrammed cells may be decreased by increased ROS levels, as suggested by the abovementioned observations of enhanced iPSC generation under hypoxic conditions. In addition, oxidative stress suppresses the ability to generate or maintain PSCs [40, 41], suggesting that ROS production induced by the reprogramming factors is unfavorable for iPSC generation. Supplementation with antioxidants, such as N-acetyl-cysteine (NAC) or vitamin C (Vc), prevents this damage, and iPSC generation is enhanced with significantly fewer de novo copy number variations (CNVs) [42] (Figure 2). Paradoxically, the depletion of ROS levels by antioxidants or Nox inhibitors in early reprogramming decreases the efficiency of iPSC generation substantially. However, excessive ROS production can also impair the efficiency of iPSC generation, and antioxidant enzyme levels are elevated in late reprogramming [43]. These data indicate that optimal ROS levels are necessary to initiate and maintain the process of efficient *in vitro* somatic cell reprogramming to pluripotency.

Interestingly, OSKM induces two different cellular fates *in vivo*: reprogramming in a subset of cells and senescence in many other neighboring cells [38, 44]. Senescent cells release paracrine factors such as interleukin-6 (IL6) to surrounding cells that promote the reprogramming and dedifferentiation [38, 39]. Thus, biological conditions associated to cellular senescence such as tissue damage and aging positively contribute to a permissive microenvironment for *in vivo* reprogramming [38, 39, 44], which seems to be contradictory to *in vitro* reprogramming. IL6 has been shown to induce ROS production in cells such as neuron, monocyte, and neutrophil, inducing a prooxidant environment [45, 46]. Paradoxically, IL6 can also induce an adaptive response to oxidative stress in normal tissues of the injury models [47, 48]. Therefore, *in vivo* OSKM-induced senescence enhances cellular plasticity, which is linked to tissue regeneration and organismal rejuvenation, although further studies are needed [49, 50].

The metabolic shift from OxPhos to glycolysis is also critical for somatic cellular reprogramming. As mentioned above, reprogrammed iPSCs have an increased dependence on glycolysis under aerobic metabolism conditions, with deliberate OxPhos suppression, similar to the Warburg effect in cancer cells. Induced pluripotency and tumorigenesis are stepwise processes that share many similarities to the immortal transformation of somatic cells [51]. Indeed, the accumulation of glycolytic intermediates is essential for rapid proliferation and minimizes ROS-induced damage in both PSCs and cancer cells [52]. Significantly, the known reprogramming factors possess oncogenic potential; for example, Oct4/Sox2 are correlated to carcinomas, and Klf4/c-Myc are well-known oncogenes [53, 54]. It has also been reported that c-Myc increases glycolysis and inhibits OxPhos, and Lin28, which is also associated with tumorigenesis and reprogramming, promotes glucose metabolism [55, 56]. In addition, the inhibition of the p53 tumor suppressor gene, which increases glycolysis as mentioned above, also enhances somatic cellular reprogramming. Similarly, PS48, which is a potent activator of PDK1; fructose 2,6-bisphosphate (Fru-2,6-P_2_); fructose 6-phosphate (F6P); 2,4-dinitrophenol (DNP); N-oxaloylglycine (NOG); quercetin; and mitochondrial inhibitors (e.g., antimycin A, rotenone, and KCN), which are involved in the metabolic transition from OxPhos to glycolysis, facilitate somatic cellular reprogramming [57–60], whereas small molecules, such as 2-deoxyglucose (2-DG), 3-bromopyruvic acid (BrPA), 6-aminonicotinamide (6-AN), oxalate, and dichloroacetate (DCA), which are associated with OxPhos, decrease the efficiency of iPSC generation [57, 60, 61] (Figure 2). These data suggest that a metabolic shift from oxidative catabolism to anaerobic glycolysis is crucial for efficient iPSC generation.

Human and mouse iPSCs are reprogrammed by the forced transduction of the same Yamanaka factors, but the cell status of iPSCs is distinctive between humans and mice. Human iPSCs are reprogrammed to a primed state similar to human ESCs, whereas mouse iPSCs are reprogrammed to a naïve state similar to mouse ESCs. Key differences between primed and naïve PSCs are in their derivation of germline competency, epigenetic states, expression patterns for pluripotency and lineage-specific genes, signaling requirements for self-renewal, and central carbon metabolism [52, 62]. In particular, naïve PSCs utilize OxPhos more than primed PSCs, which are dependent almost entirely on glycolysis [62, 63]. It remains unclear whether this difference is similar to *in vivo* situations in which embryos first use mitochondrial OxPhos but then switch to anaerobic glycolysis after implantation [52]. Current studies suggest that the metabolic shift in PSCs relies on the culture conditions [64] or the pluripotency factors that are involved in regulating the epigenetic machinery to modulate the naïve and primed pluripotency states [65, 66]. Thus, metabolic reprogramming to the pluripotent substates of PSCs may require a fine balance between the extrinsic environment containing nutrients and/or oxygen levels and the intrinsic needs mediated by the pluripotency factors [52]; however, the mechanism underlying PSC metabolic reprogramming remains largely unknown.

## 3. Oxidative Stress in HSCs

HSCs are a type of adult stem cells that undergo hematopoiesis to replenish mature blood lineages throughout an organism's lifetime [67]. For many decades, HSCs were used for treating hematological and immune diseases. However, their limited number prevents the more reliable and broader application of HSC-based therapies, and many attempts to propagate HSCs *in vitro* have failed, primarily because self-renewal and *in vivo* regenerative capacity are lost rapidly in culture [68]. Thus, genetic analyses using mutant animal model have identified essential regulators, and transcriptome, epigenome, and proteome studies have provided important insights into HSC biology [69, 70].

**Figure 3:**
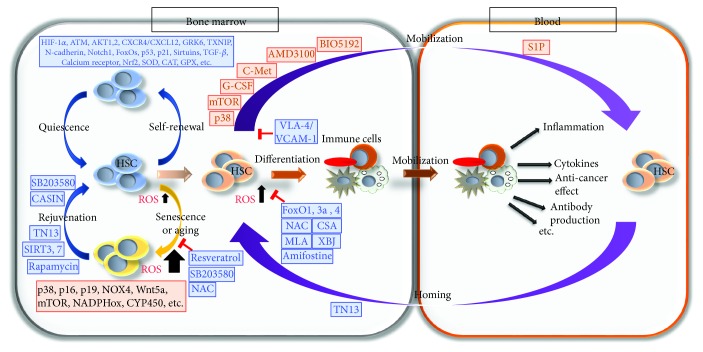
Critical regulators and pharmacological regulation of oxidative stress in HSCs. Schematic diagram illustrating the functional role of signaling proteins and pharmacological agents in the regulation of ROS in HSCs. Blue-colored proteins or agents generally reduce ROS level in HSCs or microenvironment; therefore, they help HSCs to maintain the balance between HSC self-renewal and differentiation, which is critical for tissue homeostasis. But orange-colored proteins and agents induce ROS level and result in cellular senescence or aging, differentiation, and mobilization in HSCs. Interestingly, SB203580, CASIN, TN13, and rapamycin rejuvenate aged HSCs.

HSCs reside in hypoxic niches in the bone marrow (BM), and this hypoxic environment presumably ensures that HSCs are protected from much of the oxidative stress and can maintain their self-renewal ability [71–73]. HSCs need to be protected from high ROS levels to avoid stem cell exhaustion; however, continuous low ROS production will lead to the lack of stem cell function. Ultimately, balanced ROS levels are crucial for maintaining the stem cell pool and host immunity during conditions of both homeostasis and stress [74]. Recent reports have suggested the crucial role of ROS in the regulation of differentiation, self-renewal, migration, and quiescence and proliferation balance in HSCs [74, 75]. Here, we introduce ROS as emerging regulators of HSC fate decision, motility, and aging and also describe the pharmacologic approaches in ROS regulation of HSCs (Figure 3).

### 3.1. Oxidative Stress in HSC Fate Decisions

Quiescent HSCs rely primarily on glycolysis for energy production, and compared with mitochondrial oxidative phosphorylation, glycolysis is much less efficient for energy production but is good for maintaining low levels of ROS in HSCs [76, 77]. Oxidative stress regulators are highly enriched in HSCs, and they activate robust oxidative stress responses to scavenge ROS [6]. Recently, many stem cell research groups have reported extensive interactions between HSCs and their niche via a variety of soluble factors, such as Wnt, BMP, TPO, IL-3, and IL-6; various adhesion molecules, including CXCL12-CXCR4 and N-cadherin; and different signaling pathways, including SCF/c-Kit, Jagged/Notch, and angiopoietin-1/Tie2 (Ang-1/Tie2); these interactions provide a special environment that supports the self-renewal and survival of HSCs and help them be quiescent [78, 79].

In the hematopoietic system, cellular ROS levels are considerably lower in HSCs than in differentiated lineage cells, and HSCs mostly remain in a quiescent state [6, 80]. Quiescent, long-term repopulating HSCs are characterized by low levels of ROS. Increased ROS levels enhance the cycling of HSCs and promote the exhaustion of the stem cell pool [81, 82]. Quiescent HSCs exhibit low metabolic rates and presumably produce less ROS, which are capable of causing oxidative damage. Hypoxia-inducible factor-1 *α* (HIF-1 *α*) is activated in HSCs and shifts cellular metabolism from mitochondrial respiration to glycolysis, thus limiting ROS production; without HIF-1 *α*, HSCs lose their ROS regulation ability and long-term repopulation capacity [74, 83]. The presence of the ataxia telangiectasia mutated (ATM) protein is required for HSC self-renewal and quiescence because it limits ROS levels. ATM-deficient mice showed a defect in HSC function that was associated with elevated ROS levels, and the repopulating capacity of Atm^−/−^ HSCs was rescued by N-acetyl-L-cysteine (NAC) treatment [81]. Foxo3a^−/−^ HSCs showed increased ROS levels and p38 MAPK activity and had defective quiescence maintenance; Foxo3a^−/−^ mice were sensitive to 5-FU-induced myelotoxic injury [84].

One research group has proven the relationship between ROS and hematopoietic differentiation. The critical role of ROS in the lineage decision of myeloid progenitors was proven, and high intracellular ROS levels were observed in granulocyte-monocyte progenitor cells. The authors also showed that intracellular ROS levels in common myeloid progenitors (CMPs) were inversely correlated with their MEP differentiation potential [85]. AKT 1 and AKT 2 double-deficient long-term HSCs (LT-HSCs) showed defects in repopulation capacity and ROS regulation. Double-deficient cells were sensitive to pharmacologic increases in ROS and showed increased differentiation capacity with BSO treatment [86]. In response to increasing levels of ROS, p38 MAPK limits the lifespan of HSCs *in vivo*, and the inhibition of p38 MAPK by SB203580 treatment rescued ROS-induced defects in the HSC repopulating capacity and HSC quiescence maintenance [82]. Disrupting the CXCR4 receptor in mice led to ROS production, p38 MAPK activation, DNA double-strand break induction, and apoptosis in HSCs. Increased ROS levels are directly responsible for the exhaustion of the HSC pool and repopulating capacity [64]. G protein-coupled receptor kinases (GRKs) are critically involved in immune responses through regulating cytokine receptors in mature leukocytes. GRK6^−/−^ mice exhibit lymphocytopenia, HSC loss, and multiple progenitor populations, thus leading to compromised lymphoid differentiation largely due to impaired HSC self-renewal. GRK6 is involved in ROS signaling, and ROS scavenger *α*-lipoic acid treatment partially rescued HSC loss [87]. Granulocyte colony-stimulating factor (G-CSF) is used to treat leukopenia induced by radiotherapy or chemotherapy in patients and can cause sustained low white blood cell counts in PB. This adverse effect is caused by G-CSF-induced HSC proliferation and differentiation, which impair HSC self-renewal and may exhaust the BM's capacity to exacerbate IR-induced LT-BM injury. Increased HSC damage was associated with increased ROS production, p38 MAPK activation, and senescence induction in HSCs [88].

Many of radioprotective drugs have been developed to protect hematopoietic injury from irradiation stress. Melatonin (N-acetyl-5-methoxytryptamine, MLT) and *α*-lipoic acid (LA) conjugated 5-methoxytryptamine-*α*-lipoic acid (MLA) decreased the levels of ROS in hematopoietic cells by inhibiting NOX4 expression under total body irradiation condition. MLA remarkably prevents radiation-induced hematopoietic syndrome [89]. Amifostine is a ROS scavenger and radioprotective drug that has been approved by the US Food and Drug Administration (FDA) and protects primitive hematopoietic progenitors against chemotherapy cytotoxicity [90, 91]. Xuebijing injection (XBJ) was a traditional Chinese medicine and also protected hematopoietic injury by decreasing ROS production via increasing glutathione (GSH) and superoxide dismutase (SOD) levels in serum [92].

Exposure to air during collection limited the yield of HSCs from BM and cord blood (CB). HSCs lost their long-term repopulating capacity, and progenitor cells were increased in BM and CB cells under nonphysiologic ambient air. To limit ROS production and HSC differentiation, they collected and handled HSCs under hypoxia (3% O_2_) condition and compared to air-harvested HSCs. Up to 5-fold greater number of HSCs were recovered by hypoxic harvest than air harvest. This phenomenon was mediated by ROS production linked to cyclophilin D (CypD), p53, and the mitochondrial permeability transition pore (MPTP). Interestingly, inhibition of CypD using cyclosporine A (CSA), a small molecule inhibitor of CypD, antagonized MPTP induction, reduced ROS, and enhanced the yield of HSCs and the efficacy of their transplantation [93].

Recently, many reports have suggested that the function of neighboring cells was crucial for ROS regulation of HSCs in BM niches. In particular, endothelial cells (ECs) are components of blood vessels and regulate trafficking and maintenance of HSCs in BM. One group has reported that arterial blood vessels were less permeable and maintained HSCs in a low ROS state, whereas the more permeable sinusoids promoted hematopoietic stem and progenitor cell (HSPC) activation and were used for leukocyte trafficking site. Increased permeability of blood vessels could increase ROS levels, migration, and differentiation of HSPCs by penetrating plasma, carrying ROS-inducing factors [94]. Most HSCs are present in perivascular locations in close contact with either sinusoids or arterioles [95]. Arterial ECs in the BM (aBMECs) created an endosteal vascular niche for nonactive quiescent HSCs, while sinusoidal ECs (sBMECs) constitute a leukocyte trafficking site or HSPC activation site. aBMECs showed lower ROS levels and higher glucose uptake and have different anatomical structure and metabolic signature as compared to sBMECs [94, 96]. ECs are exposed to oxygen in the blood and have developed to scavenge excessive ROS and rely mainly on glycolysis to avoid ROS production via oxidative phosphorylation [94, 97, 98]. Glycolysis in ECs may enable them to regulate ROS levels in cells and their surroundings and contribute to serve an ideal site for HSC maintenance in BM. Another neighboring cells including megakaryocytes (MKs) and nonmyelinating Schwann cells secrete transforming growth factor *β* (TGF-*β*), which is known as a niche factor to regulate HSC dormancy in BM niche [99, 100].

Overall, the importance of ROS as a critical regulator of HSC quiescence and differentiation was revealed by *in vitro* and *in vivo* signaling pathway studies and pharmacological challenges (Figure 3).

### 3.2. Oxidative Stress in HSC Motility

HSCs reside in the BM and can migrate out of the BM to the peripheral blood (PB) under stress conditions as a part of the host defense and repair mechanisms [68, 74]. HSC movement from the osteoblastic niche to the vascular niche or PB is regulated by the ROS levels in HSCs. One research group divided HSCs into ROS^low^ and ROS^high^ populations and then analyzed their functional differences. The ROS^low^ population showed higher quiescence, self-renewal potential, and calcium receptor, N-cadherin, Notch1, and p21 levels and resided in the low-oxygen osteoblastic niche; however, the ROS^high^ population showed significant HSC exhaustion after serial transplantation and p38 MAPK and mammalian target of rapamycin (mTOR) activation and resided in the high-oxygenic vascular niche. Pharmacologic inhibition of the p38 and mTOR pathways by SB203580 and rapamycin restored the functions of ROS^high^ HSCs [72].

G-CSF could mobilize hematopoietic cells in large numbers from the marrow into the circulation, with increased progenitor cells of all lineages detected in the spleens of G-CSF-treated mice [101]. Animal studies indicated that hematopoietic progenitors lacking G-CSFR were mobilized with an efficiency equivalent to those expressing the receptor. However, in the mice in which all hematopoietic cells lacked G-CSFR, these cells completely failed to mobilize. The response of hematopoietic cells to G-CSF is essential for HSC mobilization and is indirect; moreover, a specific response of individual HSCs to G-CSF is not required [101, 102]. One group has reported that G-CSF induces c-Met expression and mobilization of hematopoietic progenitor cells. G-CSF administration causes transient upregulation of stromal cell-derived factor-1 (SDF-1) and subsequently activates CXC chemokine receptor-4 (CXCR4) signaling for hepatocyte growth factor (HGF) production. HGF binds to c-Met and thus activates c-Met signaling to regulate mTOR/FOXO3a signaling pathway. Ultimately, this signaling causes ROS production and promotes hematopoietic stem and progenitor cell egress out of the BM [103].

CXCL12 is a cytokine secreted by osteoblasts, endothelial cells, and reticular mesenchymal stem and progenitor cells; in addition, CXCL12 induces active stem and progenitor cell migration and mobilization that is increased by ROS, JNK, and MMP9. However, cell surface, membrane-bound CXCL12 is essential for stem cell quiescence, retention, and self-renewal when presented by the BM stroma [104]. Elevated ROS levels promote CXCL12 secretion and then induce HSC mobilization [74]. CXCR4 is a major receptor of CXCL12 and is also regulated by oxidative stress. ROS regulate nuclear factor- (erythroid-derived 2-) related factor 2 (Nrf2) activity, and Nrf2 induces CXCR4 expression by acting directly on the CXCR4 promoter [105]. Steady-state CXCL12-CXCR4 interactions are essential for maintaining the stem cells in a quiescent nonmotile, ROS^low^ mode, suggesting that CXCL12 signaling can limit ROS levels [74]. The CXCR4 antagonist AMD3100 was first approved in 2008 by the US Food and Drug Administration (FDA) for use in combination with G-CSF to mobilize HSCs; now, AMD3100 is commonly used worldwide for this purpose. CXCR4 antagonists mobilize HSCs by blocking the retentive activity of CXCL12 [101].

Vascular cell adhesion molecule 1 (VCAM-1) binds to integrin alpha-4 (VLA-4), which is expressed by osteoblasts, and VCAM-1 binds to VLA-4 on endothelial cells. ROS are involved in modulating endothelial cell function to promote VCAM-1-dependent lymphocyte migration [106]. The VLA-4/VCAM1 adhesive interaction is disrupted during G-CSF-induced HSC mobilization [101]. A small molecule inhibitor of VLA-4 binding, BIO5192, has been developed and, as anticipated, increases the degree of mobilization induced by G-CSF in mice [107].

The bioactive lipid sphingosine 1-phosphate (S1P) is a chemo-attractant for hematopoietic cells, including HSCs, and this activity is mediated by a series of G-protein-coupled receptors, S1P1–S1P5, with S1P1 being the principal receptor on HSCs [108]. S1P is present at high concentrations in plasma and low concentrations in tissues, including the bone marrow, providing an appropriately directed gradient. Amplifying the S1P gradient between the blood and the BM provides a potential mechanism to increase HSC trafficking into the peripheral blood [101, 109]. Altogether, HSC motility is regulated by the ROS levels in HSCs or the BM microenvironment (Figure 3).

### 3.3. Oxidative Stress in HSC Aging

Organ aging is linked to the aging-associated decline in somatic stem cell function in various animal model systems. HSC aging is driven by intrinsic and extrinsic factors linked to the impaired self-renewal and regeneration of lineage cells. Defining the mechanisms regulating the process of aging is important for understanding aging-associated disease and promoting a longer and healthier lifespan [110–112]. Recent advances in HSC aging studies have reached a consensus in the phenotypes of aged HSCs. The number of HSCs increases in both mice and humans, and there are two- to tenfold more HSCs present in aged BM than in young BM [113, 114]. In serial transplantation assays, aged HSCs exhibit decreased repopulation capacity as a consequence of lower long-term self-renewal capacity and heightened replicative stress on cell cycling and decreased ribosomal biogenesis [115]. Additionally, aged HSCs lose their homing ability to the BM, and young and aged HSCs occupy distinct niches within the BM. Aged HSCs exhibit impaired adhesion to stromal cells and can then better mobilize into the PB [114, 116]. Myeloid genes are upregulated in aged HSCs, which is consistent with their myeloid bias [114]. Recently, numerous studies have aimed to prove the causal roles of ROS in HSC aging in various model systems. HSCs are relatively sensitive to oxidative stress because they reside in a hypoxic niche and are maintained in a quiescent state. A moderate increase in ROS levels can induce self-renewal and differentiation defects in HSCs via inducing HSC senescence, which causes premature HSC aging [117]. Therefore, the induction of HSC senescence resulting from increased ROS production has been implicated in the pathogenesis of BM suppression under various pathological conditions [79, 118].

In particular, DNA damage responses and increased ROS levels have been causatively attributed to HSC aging [110]. DNA damage constantly arises from DNA replication errors, spontaneous chemical reactions, and assaults from external or metabolism-derived agents. Endogenous sources of DNA damage include replication and recombination errors, spontaneous hydrolysis, and reactive metabolites, such as ROS, created as by-products of cellular metabolism [81, 114]. ATM is involved in a DNA damage checkpoint and regulates HSC self-renewal. ATM-deficient mice showed bone marrow failure after 24 weeks of age due to a functional decrease in HSCs resulting from increased ROS levels. The increase in ROS levels led to the activation of p38 mitogen-activated protein kinase (MAPK), which in turn caused the upregulation of the cyclin-dependent kinase (CDK) inhibitors p16Ink4a and p19Arf. NAC treatment restored the repopulation capacity of Atm^−/−^ HSCs, resulting in the prevention of bone marrow failure. Inducing p16INK4a and p19Arf in response to increased ROS levels might lead to cellular senescence in Atm^−/−^ HSCs. The self-renewal capacity and cellular senescence of HSCs may depend on the ATM-mediated inhibition of oxidative stress [81, 119].

Mice with conditional Foxo1, Foxo3a, and Foxo4 knockout showed myeloid lineage expansion and lymphoid developmental abnormalities, as well as a marked decrease in the lineage-negative Sca-1^+^, c-Kit^+^ (LSK) compartment, and defective long-term repopulating activity that correlated with increased cell cycling and apoptosis in HSCs. FoxO-deficient HSCs also showed a marked increase in ROS levels compared with wild-type HSCs. *In vivo* treatment with NAC resulted in the reversion of the FoxO-deficient HSC phenotypes [120]. Total body irradiation (TBI) induces long-term BM suppression in part by inducing HSC senescence through NADPH oxidase 4- (NOX4-) derived ROS. Treatment with 3,5,4′-trihydroxy-trans-stilbene (resveratrol), a potent antioxidant and putative activator of SIRT1, significantly inhibited the TBI-induced increase in ROS production in HSCs and ameliorated TBI-induced long-term BM injury by inhibiting radiation-induced chronic oxidative stress and senescence in HSCs [118]. SIRT3 is a mammalian sirtuin that regulates the global acetylation of mitochondrial proteins and reduces oxidative stress. SIRT3 is highly enriched in HSCs and is suppressed in differentiated hematopoietic cells. SIRT3 is dispensable for HSC maintenance and tissue homeostasis at a young age and under homeostatic conditions, but it is essential under stress and at an old age. Upregulating SIRT3 improves the regenerative capacity of aged HSCs. It has been suggested that SIRT3 regulates mitochondrial metabolic homeostasis and reduces ROS in HSCs; additionally, aging-associated degeneration can be reversed by sirtuins [121].

Aged HSCs showed an increase in intracellular superoxide anion (1.4-fold), hydrogen peroxide (2-fold), nitric oxide (1.6-fold), and peroxynitrite/hidroxil (2.6-fold) levels compared with young cells. Mitochondria and NADPHox were the major sources of ROS production. CYP450 contributed in middle and aged mice, and only xanthine oxidase contributed in aged mice; DNA damage and apoptosis were increased in the middle (4.2- and 2-fold, respectively) and aged (6- and 4-fold, respectively) mice, and aged mice exhibited significantly shorter telomere lengths [122]. We have found that thioredoxin-interacting protein (TXNIP) regulates intracellular ROS in HSCs by regulating p53 activity via direct interaction. TXNIP-deficient old mice exhibited elevated ROS levels in HSCs and showed a reduction in the hematopoietic cell population. TXNIP-deficient mice were more sensitive to oxidative stress. TXNIP interacted with the p53 protein and induced p53 transcriptional activity to upregulate antioxidant genes. Transducing TXNIP or p53 into Txnip^−/−^ bone marrow cells rescued the HSC frequency and greatly increased survival in mice following oxidative stress [123].

Recently, HSC aging studies have reported the concept of rejuvenation in animal models. One report has shown that prolonged fasting regulates IGF-1/PKA signaling and rejuvenates the aging-associated phenotypes including myeloid bias, reducing long-term repopulation capacity of aged HSCs [110, 124]. Mammalian target of rapamycin (mTOR) activity is increased in aged HSCs. To induce mTOR signaling, they deleted Tsc1, which encodes tuberous sclerosis complex (TSC) protein 1, leading to constitutive activation of mTOR in HSCs. TSC^−/−^ HSCs showed higher expression of aging-associated genes including p16, p19, and p21 and the reductions in hematopoiesis and in lymphopoiesis. Inhibition of mTOR activity by rapamycin enhances the lifespan of aged mice and the repopulation capacity of aged HSCs [125]. Mohrin et al. have reported the interaction between sirtuin 7 (SIRT7) and nuclear respiratory factor 1 (NRF1). NRF1 recruited SIRT7 to the proximal promoters of genes encoding mitochondrial ribosomal proteins (mRPs) and mitochondrial translation factors (mTFs). SIRT7 repressed the expression of mRPs and mTFs. SIRT7^−/−^ HSCs showed an increase in proliferation and displayed a 40% reduction in their ability to reconstitute the hematopoietic system of recipient mice and showed myeloid-biased differentiation. SIRT7 upregulation improved the regenerative capacity of aged HSCs [126]. Another groups have reported that aged HSCs showed higher expression of Wnt5a and they have showed the rejuvenation of aged HSCs by inhibiting Cdc42 activity using a specific inhibitor of Cdc42 (CASIN) [127].

Recently, we have found that TXNIP regulates the aging of HSCs by inhibiting p38 MAPK activity via direct interaction. In addition, a TXNIP-derived peptide inhibits p38 MAPK activity and rejuvenates aged HSCs by reducing ROS levels. The TXNIP/p38 MAPK axis regulated the aging of HSCs by causing a higher frequency of long-term HSCs, lineage skewing, a decrease in engraftment, an increase in ROS levels, and a loss of Cdc42 polarity. A cell-penetrating peptide- (CPP-) conjugated peptide (TN13) derived from the TXNIP-p38 interaction motif inhibited p38 activity in HSCs *in vitro* and *in vivo*, rescued homing ability, and rejuvenated aged HSCs. We have suggested that the TXNIP-p38 axis regulates HSC aging and have proven the pharmacologic potential of TN13 to rejuvenate aged HSCs [128].

From HSC aging studies, we could find that the increased ROS levels induced HSC aging; however, it could be reversible by reducing ROS using rejuvenating agents (Figure 3).

## 4. Conclusion

Here, we have introduced that oxidative stress plays a critical role as a regulator of stem cell fate decision and have described the defined mechanisms of oxidative stress regulation in stem cells. ROS regulate physiological and biological functions in cellular processes and are tightly regulated by antioxidant enzymes and modulators under normal physiological conditions. These reports have shown that the balance between stem cell self-renewal and differentiation is critical for tissue homeostasis throughout an organism's lifespan, and this balance is regulated by ROS in embryonic and adult stem cells. Oxidative stress is regulated by intrinsic or extrinsic pathways and regulates proliferation, differentiation, genomic mutation, aging, and apoptosis of stem cells. Interestingly, many of the dysregulated functions of stem cells under oxidative stress were reversible or rescued by targeting critical signaling pathways using pharmacological approaches or overexpression of specific genes. In this review, we have discussed the sources and regulation mechanisms of oxidative stress and have suggested the possibility or impact of pharmacological regulation of ROS in stem cells for regenerative medicine or clinical trials. However, stem cell research is faced with ethical and political controversies and limitations for human or animal model studies. Therefore, we need to develop new model systems to replace animal models or human primary cells. Recently, iPSCs and organoid-based three dimensional (3D) cell culture and ESC-derived HSCs are developed to replace animals or primary cells. In the future, stem cell research will be replaced by these kinds of model systems.

## Abbreviations

ATM: Ataxia telangiectasia mutated
CXCR4: CXC chemokine receptor-4
ESC: Embryonic stem cell
FoxO1: Forkhead box O 1
G-CSF: Granulocyte colony-stimulating factor
HGF: Hepatocyte growth factor
HIF-1*α*: Hypoxia-inducible factor-1*α*
HSC: Hematopoietic stem cell
iPSCs: Induced pluripotent stem cells
Klf4: Kruppel-like factor 4
mTOR: Mammalian target of rapamycin
NAC: N-acetyl-L-cysteine
Nox4: NADPH oxidase 4
Oct4: Octamer-binding transcription factor-4
OxPhos: Oxidative phosphorylation
PSCs: Pluripotent stem cells
ROS: Reactive oxygen species
SDF-1: Stromal cell-derived factor-1
SIRT1: Sirtuin 1
Sox2: Sex-determining region Y-box 2
TRX: Thioredoxin
UCP2: Uncoupling protein 2
VSMCs: Vascular smooth muscle cells.
